# Oxygen dissociation curve inflection point during incremental exercise: a trigger for the Bohr effect

**DOI:** 10.1007/s00424-025-03100-9

**Published:** 2025-07-10

**Authors:** Holger H. Burchert, William W. Stringer, Ranjan K. Dash

**Affiliations:** 1 Department of Sport, Exercise and Health, University of Basel, Basel, Canton Basel, Switzerland; 2 Department of Medicine, The Lundquist Institute for Biomedical Innovation at Harbor-UCLA Medical Center, Torrance, CA USA; 3 Departments of Biomedical Engineering and Physiology, Medical College of Wisconsin, Milwaukee, WI USA

**Keywords:** Allosteric regulation, Anaerobic threshold, Cooperative oxygen binding, Haldane effect, Hill equation, Oxygen equilibrium curve

## Abstract

We previously hypothesized that the inflection point of the oxygen dissociation curve (ODC) is linked to the gas exchange threshold (GET) during cardiopulmonary exercise testing. This hypothesis was supported by femoral venous blood gas data sampled during constant exercise below and above the GET, which showed that the ODC shifts rightward at the GET. What had gone unnoticed since these original observations in 1994 was that this rightward shift begins slightly earlier, precisely when the oxygen saturation crosses the ODC inflection point. To investigate this phenomenon, we analyzed the 1994 femoral venous blood gas data obtained during cardiopulmonary exercise testing using a modern validated mechanistic biochemical model of oxygen (O_2_), carbon dioxide (CO_2_), and proton binding to hemoglobin (Hb). We constructed the ODC for each data point, as well as the in vivo ODC—a composite curve reflecting changes in dynamic blood chemistry during exercise—to assess its alignment with the GET. The model revealed that, at the in vitro ODC inflection point (36% O_2_Hb saturation), the amounts of CO_2_ bound to Hb equalized with HbNH_3_^+^ eventually predominating. This equilibrium apparently triggered the Bohr shift, steepening the in vivo ODC to improve O_2_ unloading to the tissues. Shortly afterwards, the in vivo ODC reached its inflection point, matching the measured GET. Our findings support that the GET is mechanistically linked to the in vivo ODC inflection point. These results highlight the physiological relevance of determining the ODC inflection point and its alignment with HbNH_3_^+^ and CO_2_ binding as critical factors in understanding ODC shifts during cardiopulmonary exercise testing.

## Introduction

Hemoglobin (Hb) is blood’s multi-purpose transport molecule. It carries oxygen (O_2_) from the lungs to the tissues and carbon dioxide (CO_2_) from the tissues to the lungs, regulated by the binding of protons (H^+^) and the molecule 2,3-diphosphoglycerate (2,3-DPG). The binding of O_2_ to Hb, described by the S-shaped O_2_ dissociation curve (ODC), is influenced by the binding of the other substances and by other physiological conditions such as temperature. A thorough understanding of these interactions of Hb is crucial for the treatment, diagnosis, and prognosis of many diseases, as well as for the development of new drugs to treat such diseases.

We recently published an article proposing that the inflection point of the ODC, defined by a change in its concavity, is related to the gas exchange threshold (GET) observed during incremental exercise [10]. This is the moment during an incremental exercise test when the linear relationship between the rate of CO_2_ volume expired ($\dot{\text V}\text{CO}_2$) and the rate of O_2_ volume consumed ($\dot{\text V}\text{O}_2$) becomes steeper [5]. The origin of the GET is understood based on the bicarbonate buffer reaction:

1 $${CO}_{2}+{H}_{2}O\stackrel{{K}_{1}^{{\prime}}}{\rightleftharpoons } {H}_{2}{CO}_{3}\stackrel{{K}_{1}^{{\prime}{\prime}}}{\rightleftharpoons } {{HCO}_{3}}^{-}+{H}^{+}$$

where the binding constants ${K}_{1}^{{\prime}}$ and ${K}_{1}^{{\prime}{\prime}}$ are as defined in Table 1. When exercise intensity reaches a certain level (GET), increasing lactic acid production and increasing ATP hydrolysis release H^+^ ions and shift the equilibrium of the above reaction to the left, towards free CO_2_. This excess CO_2_ is then expired on top of the metabolically produced CO_2_, thus increasing the $\dot{\text V}\text{CO}_2$ vs $\dot{\text V}\text{O}_2$slope [5, 38]. It occurred to us that the GET might be related to the ODC inflection point upon re-examining the invasive cardiopulmonary exercise testing data published by Stringer et al. in 1997 [10, 49]. Stringer et al. had conducted invasive incremental exercise testing to exhaustion (i.e., to maximal rate of O_2_ volume consumed = $\dot{\text V}\text{O}_2$max) with simultaneous arterial and mixed-venous blood sampling. We highlighted that mixed-venous O_2_ content decreased in an S-shaped manner with increasing exercise intensity [10]. The inflection point of this in vivo curve coincided closely with the GET [10]. In fact, such exercise tests are the most physiological way to map the in vivo ODC [19], as they are a real-life scenario for Hb to unload and load O_2_, CO_2_, H^+^, and 2,3-DPG.

**Table 1:** Model parameter values used in calculations. Unless otherwise noted, the kinetic parameter values are at *T* = 37 °C

Parameter	Definition	Value	Unit
${K}_{1}^{{\prime}}$	Equilibrium constant for the CO_2_ hydration reaction: CO_2_ + H_2_O$\rightleftharpoons$H_2_CO_3_	1.4 × 10^−3^	Unitless
${K}_{1}^{{\prime}{\prime}}$	Ionization constant of H_2_CO_3_	5.5 × 10^−4^	M
${K}_{2}^{{\prime}}$	Equilibrium constant for the CO_2_ and HbNH_2_ binding reaction: CO_2_ + HbNH_2_ $\rightleftharpoons$HbNHCOOH	21.5	M^−1^
${K}_{2}^{{\prime}{\prime}}$	Ionization constant of HbNHCOOH	1 × 10^−6^	M
${K}_{3}^{{\prime}}$	Equilibrium constant for the CO_2_ and O_2_HbNH_2_ binding reaction: CO_2_ + O_2_HbNH_2_ $\rightleftharpoons$O_2_HbNHCOOH	11.3	M^−1^
${K}_{3}^{{\prime}{\prime}}$	Ionization constant of O_2_HbNHCOOH	1 × 10^−6^	M
${K}_{5}^{{\prime}{\prime}}$	Ionization constant of HbNH_3_^+^	2.4 × 10^−8^	M
${K}_{6}^{{\prime}{\prime}}$	Ionization constant of O_2_HbNH_3_^+^	1.2 × 10^−8^	M
*α*	Parameter governing ${PO}_{2}$-dependent variable Hill coefficient *nH* with variable cooperativity hypothesis for O_2_ binding to Hb	2.8	Unitless
*β*	Parameter governing ${PO}_{2}$-dependent variable Hill coefficient *nH* with variable cooperativity hypothesis for O_2_ binding to Hb	1.20	Unitless
*γ*	Parameter governing ${PO}_{2}$-dependent variable Hill coefficient *nH* with variable cooperativity hypothesis for O_2_ binding to Hb	29.2	mmHg
*P*_50,S_	Level of ${PO}_{2}$ at which Hb is 50% saturated by O_2_ at standard physiological levels of ${PCO}_{2}$, pH_rbc_, [DPG]_rbc_, and *T*	26.8	mmHg
*PO*_2,S_	Standard partial pressure of O_2_ in blood	100	mmHg
*PCO*_2,S_	Standard partial pressure of CO_2_ in blood	40	mmHg
pH_pl,S_	Standard pH in plasma	7.4	Unitless
[DPG]_rbc,S_	Standard 2,3-DPG concentration in RBCs	4.65 × 10^−3^	M
*T*_S_	Standard temperature of blood	37	°C

**Fig. 1:**
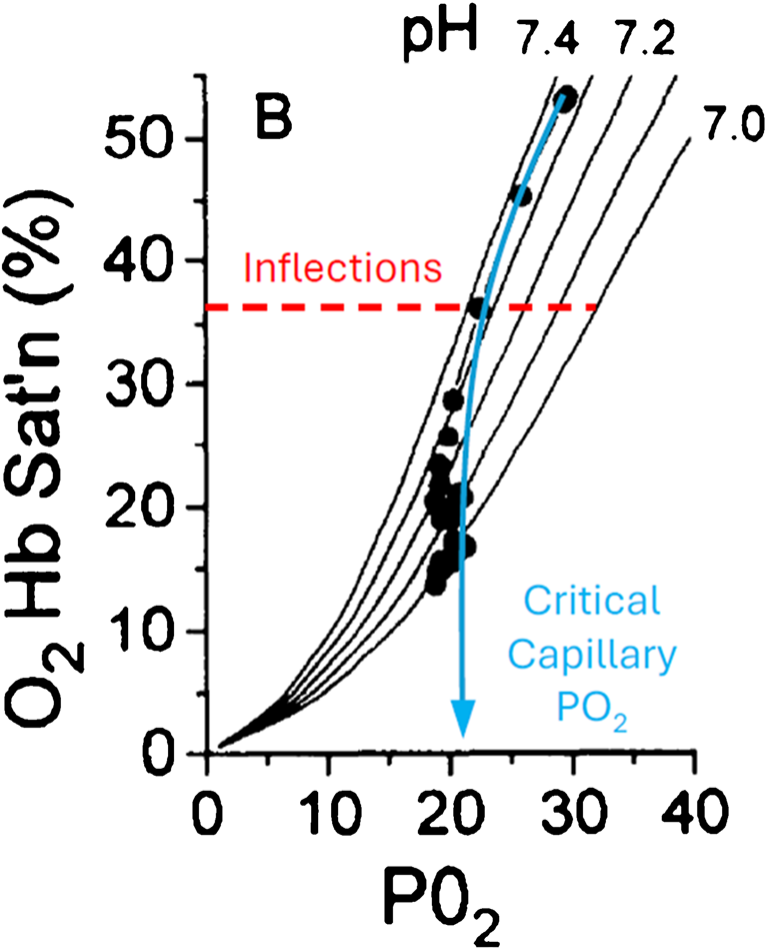
Fig. 4B from Stringer et al. (1994) [48], adapted with permission, showing group mean femoral vein O_2_Hb saturation vs. PO_2_ during 6-min constant work rate exercise at an intensity of GET $\dot{\text V}\text{O}_2$ plus 75% of the $\dot{\text V}\text{O}_2$ difference between GET and $\dot{\text V}\text{O}_2$max. Blood was sampled at rest, every 5 s during the first 120 s of exercise (using a computer-driven anaerobic collector with time correction for dead space of the catheter), and then every 30 s for the remainder of the 6 min of exercise. Plasma pH lines were calculated using the Severinghaus model [44]. The blue line shows the sample’s pathway, while the red line marks 36% O_2_Hb saturation, the O_2_Hb saturation of the inflection point being fixed in the Severinghaus model irrespective of curve shifts [10]

A second argument we presented was an article that Stringer et al. had published 3 years earlier in 1994 [48]. At that time, they collected femoral venous blood during incremental exercise testing as well as constant work rate exercise below and above GET. They observed that oxyhemoglobin (O_2_Hb) desaturation followed the ODC until the moment GET was reached, which occurred at a partial pressure of O_2_ (PO_2_) of ~ 21 mmHg. The consequential rise in H^+^ began to shift the ODC, as calculated by the Severinghaus model [44], to the right. By this, the down leftwards pathway of the lower convex part of the ODC was countered so that the PO_2_ stayed at 21 mmHg for the rest of the exercise (Fig. 1).

The authors called this the critical capillary PO_2_ [21, 48]. What remained unseen was that the moment O_2_Hb desaturation started to depart from the lower convex part of the ODC occurred slightly before the critical capillary PO_2_, coinciding precisely with the inflection point of the ODC that they were superimposing, as highlighted in Fig. 1 [10]. In fact, Wittenberg and Wittenberg, who had first postulated such a “critical capillary PO_2_“ of around 20 mmHg noted in 1970 that this would be close to the “lower inflection” of the ODC [55, 56]. However, no further investigation of this coincidence has been carried out. The purpose of the present work was therefore to recreate Fig. 1 for the incremental exercise study data published by Stringer et al. in 1994 and then explore the biochemical mechanisms that might explain how the GET is related to the inflection point of the ODC and thereby gaining a deeper mechanistic understanding of Hb transport function in blood during cardiopulmonary exercise testing.

## Methods

### Data extraction and regression analysis

**Fig. 2:**
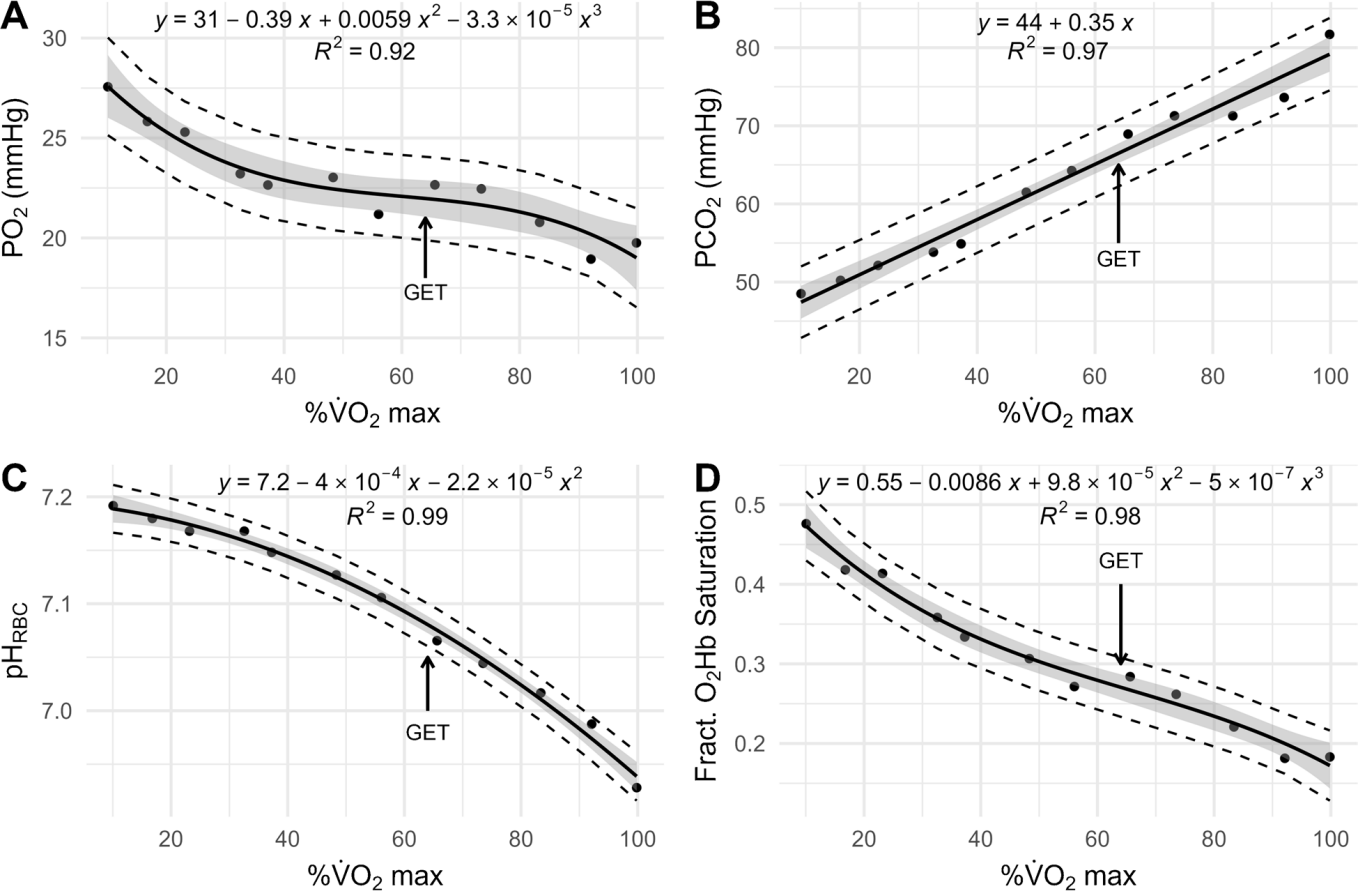
Redrawn Fig. 1A,D,C,B from Stringer et al. 1994 with permission [48] showing group mean femoral vein PO_2_, PCO_2_, pH, and O_2_Hb saturation (*n* = 12) from the incremental exercise testing as functions of %$\dot{\text V}\text{O}_2$max. Blood was sampled at rest, unloaded cycling, and each minute during increasing work rate exercise. Arrows mark the gas exchange threshold (GET = 64% $\dot{\text V}\text{O}_2$max). Gray shading shows 95% confidence intervals, and dashed lines indicate 95% prediction intervals

The original data were collected by Stringer et al. from five healthy, nonsmoking male subjects in an incremental work rate protocol, as previously described [48]. The subjects had a mean (± standard deviation) age of 25 (± 6) years, height of 179 (± 4.2) cm, weight of 72 (± 4.9) kg, and $\dot{\text V}\text{O}_2$max of 3.91 (± 0.68) L/min. The study had been conducted in accordance with the Declaration of Helsinki and the Common Rule. All procedures had been institutionally approved, and informed consent had been obtained from each participant [48]. Stringer et al. had plotted the *n* = 12 pooled, mean femoral vein PO_2_, partial pressure for carbon dioxide (PCO_2_), plasma pH, and O_2_Hb saturation as a function of %$\dot{\text V}\text{O}_2$max for their five participants in their 1994 article (redrawn Fig. 2 A-D) [48]. The mean lactic acidosis threshold (LAT/$\dot{\text V}\text{O}_2$max) occurred at 64 (± 7)% $\dot{\text V}\text{O}_2$max, corresponding to the later reference to GET = 64% $\dot{\text V}\text{O}_2$max in Fig. 2.

To obtain accurate numerical data from these plots, we followed the Cochrane Handbook’s recommendation to use software instead of manual measurement, selecting WebPlotDigitizer (RRID:SCR_013996) version 4.5 online: https://apps.automeris.io/wpd4/ [39]. The numerical values were obtained by marking each point individually. This was done by a single investigator to minimize bias. We then fitted the femoral vein PO_2_, PCO_2_, pH, and O_2_Hb saturation as function of %$\dot{\text V}\text{O}_2$max that Stringer et al. had measured with appropriate regression fit functions (Fig. 2). For O_2_Hb saturation and PO_2_, we decided to fit as high as 3rd order polynomial functions, allowing for S-shaped patterns (Fig. 2A,D). This was done because it is already known that these relationships follow S-shaped patterns [10, 49, 52, 53].

#### Data analysis with Hb and O_2_-CO_2_-H^+ ^binding modelling

To recreate Fig. 1 for the incremental exercise testing data, we used the Dash et al. model for O_2_ and CO_2_ saturations of Hb [13]. In brief, Hb consists of four heme-amino acid chains—two alpha and two beta chains. Each chain includes a heme group that binds an O_2_ molecule and has a terminal amino group, -NH_2_, which can bind a CO_2_ molecule to form an ionizable carbamino terminus, -NHCOOH.

The model is based on the biochemical reactions inside red blood cells (RBCs), whereby it enables, among other things, the calculation of the ODC and the CO_2_ dissociation curve (CDC) derived from Eqs. (2)–(6):

2 $${CO}_{2}+{HbNH}_{2}\stackrel{{K}_{2}^{{\prime}}}{\rightleftharpoons } HbNHCOOH\stackrel{{K}_{2}^{{\prime}{\prime}}}{\rightleftharpoons } {HbNHCOO}^{-}+{H}^{+}$$

3 $${CO}_{2}+{{O}_{2}HbNH}_{2}\stackrel{{K}_{3}^{{\prime}}}{\rightleftharpoons } {O}_{2}HbNHCOOH\stackrel{{K}_{3}^{{\prime}{\prime}}}{\rightleftharpoons } {{O}_{2}HbNHCOO}^{-}+{H}^{+}$$

4 $${O}_{2}+{HbNH}_{2}\stackrel{{K}_{4}^{{\prime}}}{\rightleftharpoons } {{O}_{2}HbNH}_{2}$$

5 $${{HbNH}_{3}}^{+}\stackrel{{K}_{5}^{{\prime}{\prime}}}{\rightleftharpoons }{HbNH}_{2}+ {H}^{+}$$

6 $${{{O}_{2}HbNH}_{3}}^{+}\stackrel{{K}_{6}^{{\prime}{\prime}}}{\rightleftharpoons }{{O}_{2}HbNH}_{2}+ {H}^{+}$$

where the binding constants *K’*s are as defined in Table 1. O_2_ and CO_2_ saturations of Hb are then calculated by:

7a,b $$S_{{HbO}_2}=\frac{K_{{HbO}_2}(\alpha_{O_2}\times PO_2)}{1+K_{{HbO}_2}(\alpha_{O_2}\times PO_2)};S_{{HbCO}_2}=\frac{K_{{HbCO}_2}(\alpha_{{CO}_2}\times P{CO}_2)}{1+K_{{HbCO}_2}(\alpha_{{CO}_2}\times PCO_2)}$$

where PO_2_ and PCO_2_ are the partial pressures of O_2_ and CO_2_ in mmHg, respectively, and ${\alpha }_{{O}_{2}}$ and ${\alpha }_{{CO}_{2}}$ are the solubilities of O_2_ and CO_2_ in water corrected for the effects of temperature:

8a $$\alpha_{O_2}=\left[1.37-1.37\times10^{-2}\left(T-37\right)+5.8\times10^{-4}\left(T-37\right)^2\right]\times\lbrack10^{-6}/W_{pl}\rbrack\;M/mmHg$$

8b $$\alpha_{{CO}_2}=\left[3.07-5.7\times10^{-2}\left(T-37\right)+2\times10^{-3}\left(T-37\right)^2\right]\times\lbrack10^{-5}/W_{pl}\rbrack\;M/mmHg$$

Here, ${W}_{pl}$ = 0.94 is the fractional water space of plasma and *T* is in degrees Celsius (°C). Thus, ${\alpha }_{{O}_{2}}$= 1.46 × 10^−6^ M/mmHg and ${\alpha }_{{CO}_{2}}$ = 3.27 × 10^−5^ M/mmHg at 37 °C. The apparent equilibrium constants of Hb with O_2_ and CO_2_ (${K}_{{HbO}_{2}}$ and ${K}_{{HbO}_{2}}$ with units of M^−1^) are based on single-step bindings of O_2_ and CO_2_ to Hb:

9a $$K_{{HbO}_2}=\frac{K_4'(K_3'{(\alpha}_{{CO}_2}\times PCO_2)\times\Phi_2+\Phi_4)}{K_2'(\alpha_{{CO}_2}\times P{CO}_2)\Phi_1+\Phi_3}$$

9b $$K_{{HbCO}_2}=\frac{K_2'\Phi_1+K_3'K_4'(\alpha_{O_2}\times PO_2)\Phi_2}{\Phi_3+K_4'(\alpha_{O_2}\times PO_2)\Phi_4}$$

The terms $\Phi_1-\Phi_4$ involving the interactions of H^+^ with Hb-bound O_2_ and CO_2_ are given by:

10a-d $${\Phi }_{1}=1+\frac{{K}_{2}^{{\prime}{\prime}}}{pH};{\Phi }_{2}=1+\frac{{K}_{3}^{{\prime}{\prime}}}{pH};{\Phi }_{3}=1+\frac{pH}{{K}_{5}^{{\prime}{\prime}}};{\Phi }_{4}=1+\frac{pH}{{K}_{6}^{{\prime}{\prime}}}$$

and $${K}_{4}^{{\prime}}$$ by:

11 $${K}_{4}^{{\prime}}=\frac{{{(\alpha }_{{O}_{2}}\times P{O}_{2})}^{nH-1}({K}_{2}^{{\prime}}{\alpha }_{{CO}_{2}}P{CO}_{2}{\Phi }_{1}+{\Phi }_{3})}{{\left({\alpha }_{{O}_{2}}{P}_{50}\right)}^{nH}({K}_{3}^{{\prime}}{\alpha }_{{CO}_{2}}P{CO}_{2}{\Phi }_{2}+{\Phi }_{4})}.$$

P_50_ was adjusted according to the following equations where the standard physiological values are denoted by the subscript “S” and are listed in Table 1.

12a $${P}_{50,\Delta pH}={P}_{50,S}-25.535\left(pH-{pH}_{S}\right)+10.646{\left(pH-{pH}_{S}\right)}^{2}-1.764{(pH-{pH}_{S})}^{3}$$

12b $${P}_{50,\Delta {CO}_{2}}={P}_{50,S}+1.273\times {10}^{-1}\left({PCO}_{2}-{PCO}_{2,S}\right)+1.083\times {10}^{-4}{({PCO}_{2}-{PCO}_{2,S})}^{2}$$

12c $${P}_{50,\Delta DPG}={P}_{50,S}+795.63\left(\left[DPG\right]-{[DPG]}_{S}\right)-19660.89{(\left[DPG\right]-{\left[DPG\right]}_{S})}^{2}$$

12d $${P}_{50,\Delta T}={P}_{50,S}+1.435\left(T-{T}_{s}\right)+4.163\times {10}^{-2}{(T-{T}_{S})}^{2}+6.86\times {10}^{-4}{(T-{T}_{S})}^{3}$$

The contribution of each physiological variable to the resulting *P*_50_ is by multiplication of normalized individual *P*_50_’s:

13 $${P}_{50}={P}_{50,S}({P}_{50,\Delta pH}/{P}_{50,S})({P}_{50,\Delta {CO}_{2}}/{P}_{50,S}) ({P}_{50,\Delta DPG}/{P}_{50,S})({P}_{50,\Delta T}/{P}_{50,S})$$

The Hill coefficient *nH* contains a variable cooperativity hypothesis with the Hill coefficient being expressed at low PO_2_ as simple exponential function of PO_2_:

14 $$nH= \alpha -\beta \times {10}^{-({PO}_{2}/\gamma )}$$

where $\alpha$, $\beta$, and $\gamma$ are the parameters that govern an apparent cooperativity of O_2_ for Hb (Table 1).

The model takes seven inputs: PO_2_, (mmHg), PCO_2_ (mmHg), intra-RBC pH, intra-RBC 2,3-DPG concentration (M), temperature (°C), Hb concentration (M), and hematocrit (Hct; volume fraction of RBCs in whole blood) and provides O_2_Hb and CO_2_Hb saturations (Eq. 7(a,b)) along with total O_2_ and CO_2_ contents of whole blood as outputs. Thus, PO_2_ and PCO_2_ (Fig. 2A,B), comprising 2 of the 7 inputs, could be directly entered into the model for the 12 samples. The Dash et al. 2016 model requires the pH value inside RBCs as input, rather than that of plasma. The model assumes that plasma and intra-RBC pH levels follow the Gibbs-Donnan equilibrium condition:

15 $${pH}_{plasma}= {pH}_{RBC}-log10(0.69)$$

where 0.69 is the Gibbs-Donnan ratio across the RBC membrane. We rearranged this equation solving for ${pH}_{RBC}$ given the ${pH}_{plasma}$ that Stringer et al. had measured (Fig. 2C). Consequently, 3 of 7 model input variables were available for each of the 12 samples. Stringer et al. assumed no temperature increase during the 6-min incremental exercise tests [41, 48]. While this may apply to body core temperature, femoral vein temperature has been shown to increase by 1 °C during 12-min incremental exercise testing [16]. We therefore increased the temperature linearly from 37 to 38 °C across the 12 samples. Therefore, 4 of 7 input variables were available for each sample. Hct and Hb levels do not affect O_2_Hb and CO_2_Hb saturation calculations in the model, so the exact values for these two inputs are irrelevant for this purpose. They are necessary for the total O_2_ and CO_2_ content calculations for the whole blood, which are not considered in the present study nor in the Stringer et al. study. Therefore, we set physiologically realistic values of 0.45 for Hct and 0.00528 M Hb concentration in RBCs, as in the Dash et al. model. Hence, 6 of the 7 input variables are now described. As Mairbäurl reviewed, 2,3-DPG is unaffected during acute exercise [6, 14, 27, 30, 37, 45, 46]. So, we initially set intra-RBC 2,3-DPG concentration to the Dash et al. model’s default value of 0.00465 M. With all 7 inputs available, we calculated O_2_Hb saturation comparing it to Stringer et al.’s measurements (Fig. 2D) via Bland–Altman analysis. This returned an average error of three percentage points, which we corrected by reducing the intra-RBC [2,3-DPG] to 0.0029 M. In summary, for the 12 pooled samples in 5 subjects, PO_2_, PCO_2_, and ${pH}_{RBC}$ were available, temperature increased linearly from 37 to 38 °C, Hct set to 0.45, Hb level within RBCs to 0.00528 M, and [2,3-DPG] set to 0.0029 M for all samples. The same inputs were used for the fitted values of PO_2_, PCO_2_, and ${pH}_{RBC}$ to generate in vivo dissociation curves (Fig. 2A–C).

To determine the effect of temperature on the results, we carried out a sensitivity analysis by fixing temperature to 37 °C as originally done by Stringer et al. and adjusted [2,3-DPG] to 0.00350 M to nullify the mean bias resulting from the Dash et al.’s model default [2,3-DPG] of 0.00465 M.

#### Calculation of dissociation curves, inflection points, and intersection

Dissociation curves were calculated by running the 7 inputs of each sample through the Dash et al. 2016 model along with PO_2_ values from 0 to 100 mmHg. To find the inflection points, we computed the first and second derivatives of the curves with respect to PO_2_ using central difference numerical differentiation. This allowed us to analyze the change in slope of the curves. The inflection point corresponds to the point where the second derivative of a curve equals zero. We used a numerical root-finding algorithm (uniroot function in R) to identify the PO_2_ value at which the second derivative crossed zero, thus determining the inflection point.

Upon plotting the ODC, CDC, we realized that the CDC and HbNH_3_^+^ dissociation curve (HDC) intersected around sample 4. To identify this PO_2_, we first applied linear interpolation to both curves (“approxfun” in R). The difference between the two interpolated functions was then calculated and used as the input for the root-finding algorithm. The root of the difference function corresponds to the PO_2_ value where the CDC and HDC intersect. Using the Dash et al.’s 2016 model, we also calculated the chemical species HbNH_2_, HbNH_3_^+^, O_2_HbNH_2_, O_2_HbNH_3_^+^, HbNHCOO^−^, O_2_HbNHCOO^−^, HbNHCOOH, O_2_HbNHCOOH, and HCO_3_^−^ and plotted them as functions of %$\dot{\text V}\text{O}_2$max to depict how the equilibria in Eqs. (1) to (6) behave during cardiopulmonary exercise testing. Since the model does not automatically return HCO_3_^−^, we stayed with the original equation Stringer et al. derived from the Siggaard-Andersen nomogram [47, 48]:

16 $${{HCO}_{3}}^{-}\left(\frac{mmol}{L}\right) ={10}^{({pH}_{plasma}-7.376\times \left(1-0.00305\times Hb\right)+0.848\times \left(1-0.0162\times Hb\right)\times log10\left({PCO}_{2}\right))}$$

where Hb is in g/dL and PCO_2_ in mmHg. All analyses were performed using R Statistical Software (The R Foundation for Statistical Computing, version 4.2.2, 2022–10–31, RRID:SCR_001905). In addition to R base, the packages ggplot2 (v3.4.4, RRID:SCR_014601), dplyr (v1.1.4, RRID:SCR_016708), tidyr (v1.3.0, RRID:SCR_017102), pracma (v2.4.4, RRID:SCR_026021), patchwork (v1.2.0, RRID:SCR_024826), cowplot (v1.1.1, RRID:SCR_018081), and ggpubr (v0.06, RRID:SCR_021139) were integrated and are available at https://cran.r-project.org/. All data, analysis code, and newly created materials used in this study are freely available for replication and further analysis. The complete dataset and code for reproducing the study’s findings, as well as details on any materials used, are freely available via the Zenodo (RRID:SCR_004129) repository (DOI: 10.5281/zenodo.15614693) [9].

## Results

**Fig. 3:**
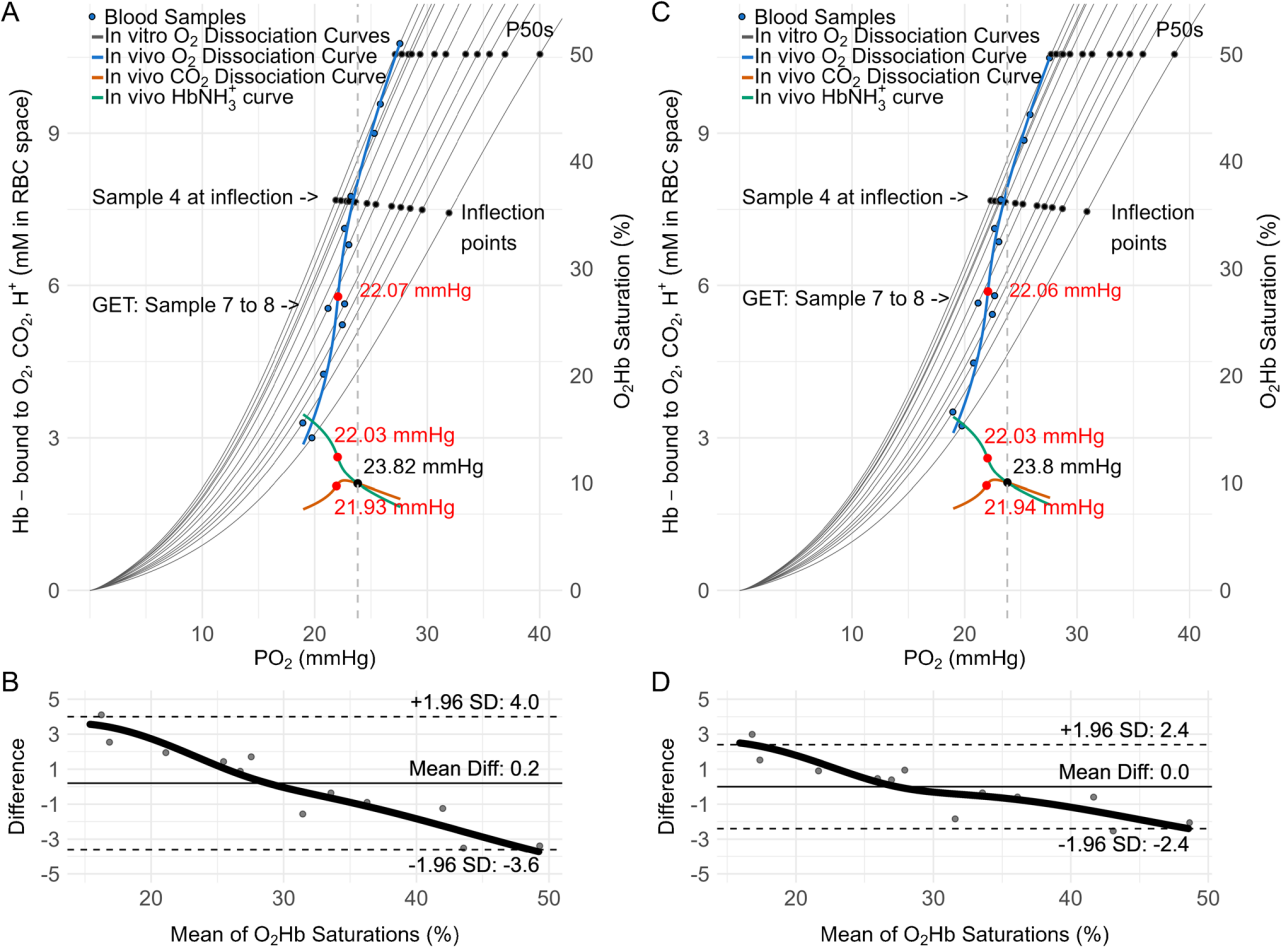
**A** Recreated Fig. 4B from Stringer et al. 1994 incremental exercise test data (herein Fig. 1) [48]. Blue dots show O_2_Hb saturation vs. PO_2_ for *n * = 12 mean samples from five participants. Gray lines are the O_2_Hb dissociation curves calculated using the Dash et al. 2016 model with each sample’s PO_2_, PCO_2_, pH, [2,3-DPG] =  0.0029 M, Hct =  0.45, and Hb =  0.00528 M (Fig. 2) and a temperature increase from 37.0 to 38.0 °C, while the blue curve represents the in vivo O_2_Hb dissociation curve based on the fitted values of Fig. 2. Black dots at 50% O_2_Hb saturation indicate the P50 for each curve, and those around 36% mark the inflection points. CO_2_Hb (orange) and HbNH_3_^+^ (green) in vivo dissociation curves are plotted with the dashed gray line marking the intersection’s alignment with sample 4 passing its in vitro ODC inflection point. **B** Bland–Altman plot comparing the O_2_Hb saturation measured by Stringer et al. with the simulated ones shown in Panel A. **C, D** The same analyses as in panels A, B but with 37.0 °C fixed and [2,3-DPG] set to 0.00350 M

In Fig. 3A, we depict the recreation of Fig. 1 for the 12 samples. For the first four blood samples, the ODC shifts very little to the right, with the samples following the curvature of the in vitro ODC. Sample four was taken the moment it passes the in vitro inflection point at 23.0 mmHg and 36% O_2_Hb saturation, thereby entering the convex part of the in vitro ODC. At almost the same PO_2_ (23.82 mmHg), the in vivo CDC and HDC intersect meaning the amounts of CO_2_ bound and HbNH_3_^+^ equalize. Now, from sample 4 to 7, the sample’s path departs from the convexity of the in vitro ODC with a larger right shift of the in vitro ODC. At the same time, the in vivo HDC starts to increase progressively while the in vivo CDC peaks and starts to decrease. Between samples 7 and 8, the GET occurred. The blue solid line is the O_2_Hb saturation for the fitted values of Fig. 2, which describes the in vivo ODC of which the inflection point was determined at 22.07 mmHg (red dot), matching GET Stringer et al. had measured between sample 7 and 8 by < 0.9 mmHg (21.2 mmHg) [48]. Closely, also the inflection points of the in vivo HDC and the CDC occur (22.03 and 21.93 mmHg, respectively). Compared to the measured and fitted O_2_Hb saturations reported by Stringer et al., those simulated saturations yielded a mean difference of 0.2 percentage points with 95% limits of agreement from –3.6 to 4.0 percentage points as shown in the corresponding Bland–Altman plot (Fig. 3B). A proportional bias with overestimation at low saturations and underestimations at high saturations is apparent.

The sensitivity analysis fixing temperature to 37 °C and [2,3-DPG] to 0.00350 M, shifted the calculated PO_2_ locations of the inflections and intersection shown in Fig. 3A by no more than 0.02 mmHg (Fig. 3C). The mean difference in O_2_Hb saturation was 0.0 percentage points, with 95% limits of agreement from − 2.4 to + 2.4 percentage points as shown in the Fig. 3D.

**Fig. 4:**
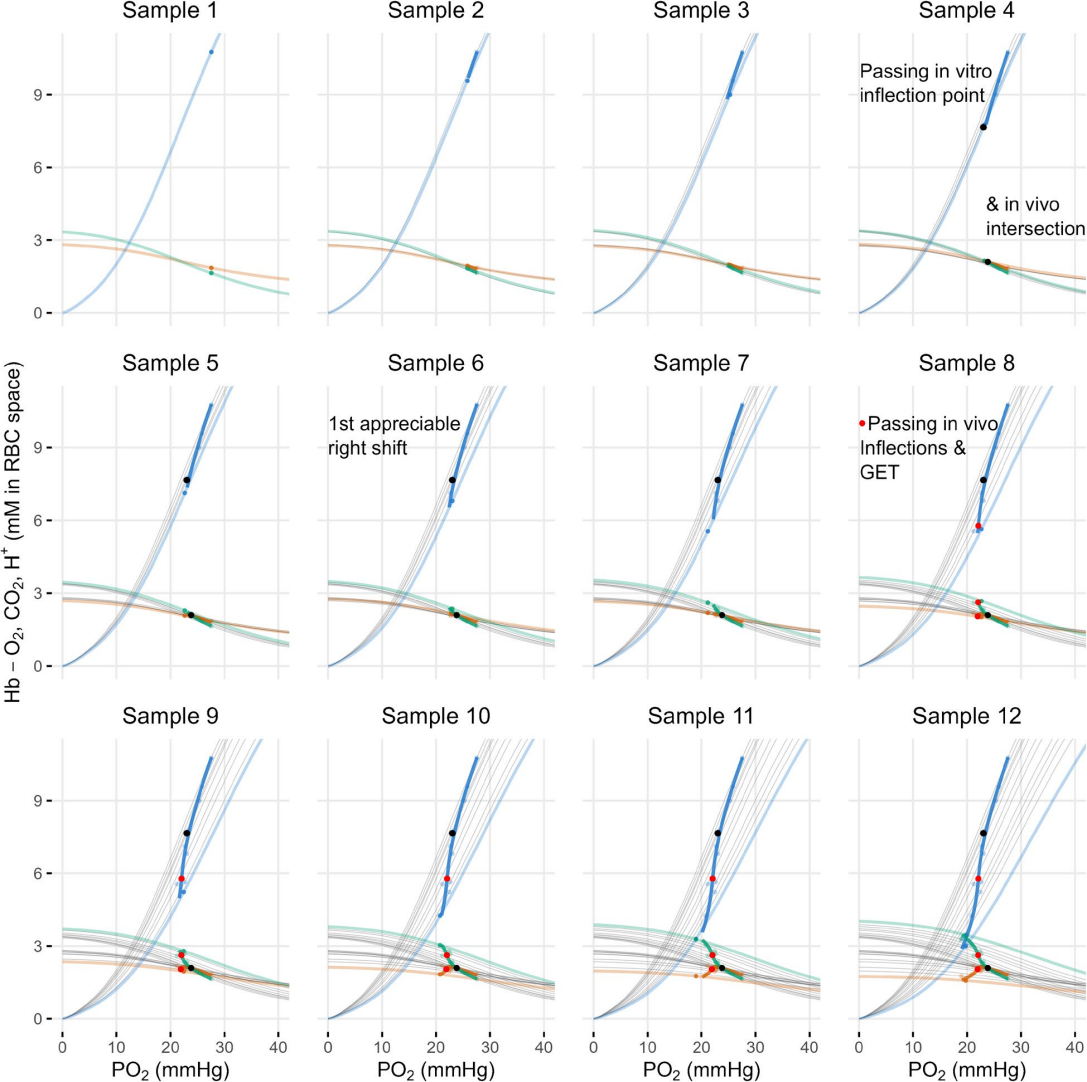
Illustration of the progression of O_2_Hb (blue), CO_2_Hb (orange), and HbNH_3_^+^ (green) across the n = 12 samples, highlighting shifts in the in vitro curves and the corresponding development of the in vivo curves. Each panel presents the current sample’s data point along with its in vitro ODC, CDC, and HDC (light-colored), all previous samples’ in vitro curves (gray), and the trace of the in vivo curves as they develop from earlier data points to the present (dark-colored)

The 12-panel plot in Fig. 4 illustrates the shifts in the in vitro ODC, CDC, and HDC across the 12 samples, along with the corresponding progression of the in vivo curves. Each panel represents an individual sample, showing how the in vitro ODC, CDC, and HDC shift as a function of PO_2_ (light-colored curves). Additionally, the in vitro dissociation curves from preceding samples (gray) are overlaid, while the dark-colored curves trace the development of the in vivo curves from previous data points to the present. By visualizing this progression, Fig. 4 shows how these stepwise changes ultimately give rise to the full set of curves presented in Fig. 3A.

**Fig. 5:**
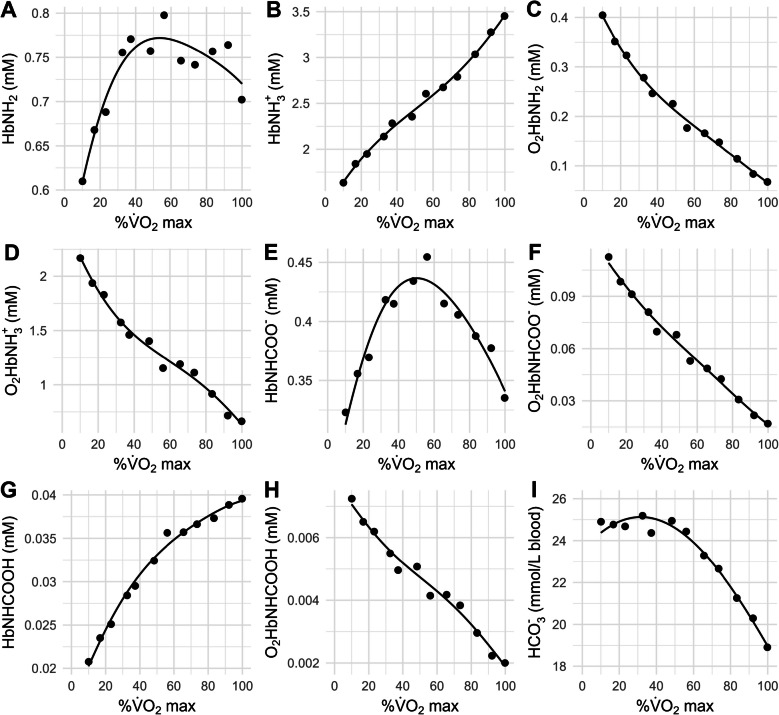
The chemical species the Dash et al. 2016 model returns for the *n *= 12 samples of Stringer et al. 1994 and their fitted values (solid lines) as functions of %$\dot{\text V}\text{O}_2$max (Fig. 2), identifying the behavior of the equilibria of Eqs. (1) to (6). Note: The physiological parameters in the Dash et al. model vary as in the experiments of Stringer et al. (Fig. 2), capturing natural interactions among variables within the system

In Fig. 5, we show the Hb-bound species as function of %$\dot{\text V}\text{O}_2$max to illustrate how the six reactions (Eqs. (1) to (6)) on which the Dash et al. 2016 model is based shift. HbNH_2_ (Eqs. 2, 4, and 5) rises first with increasing %$\dot{\text V}\text{O}_2$max before starting to decline (Fig. 5A). Its protonated counterpart, HbNH_3_^+^ (Eq. 5), increases throughout (Fig. 5B) due to decreases in pH_RBC_ or increases in intra-RBC [H^+^] (Fig. 2C). The oxygenated forms O_2_HbNH_2_ and O_2_HbNH_3_^+^ decline throughout (Fig. 5C,D) due to decreases in PO_2_ (Fig. 2A). Interestingly, HbNHCOO^−^ initially increases, then peaks, and then decreases with increasing %$\dot{\text V}\text{O}_2$max (Fig. 5E). O_2_HbNHCOO^−^ declines throughout the exercise test (Fig. 5F). HbNHCOOH increases throughout (Fig. 5G), while O_2_HbNHCOOH decreases throughout (Fig. 5H). HCO_3_^−^ is initially stable but then decreases with increasing %$\dot{\text V}\text{O}_2$max (Fig. 5I).

## Discussion

In this study, we show that the Bohr shift is triggered at the in vitro ODC inflection point by a coordinated interplay of CO_2_ and H⁺ binding to Hb. We also identify the in vivo ODC inflection point during cardiopulmonary exercise as a physiologically meaningful and non-invasively detectable event, marked by the GET, thereby directly linking Hb function to systemic threshold behavior providing new mechanistic insight into its origin.

We believe that our results support the observations of Malte et al. [28, 29]. The authors found that the Bohr-Haldane effect is of paramount importance in setting the shape and position of the ODC by highlighting an important but often overlooked detail: it is not the H^+^ in free solution that affect the O_2_ affinity of Hb per se, but rather the H^+^ bound to Bohr groups of Hb that do. Consequently, when constructing a typical in ODC at a certain PCO_2_ and pH level, any given PO_2_ inherently involves Hb that has already bound H^+^ and CO_2_. This is crucial, as CO_2_ and H^+^ binding affect O_2_ binding (Bohr effect), while O_2_ binding influences CO_2_ and H^+^ binding (Haldane effect), with the two effects being quantitatively equal at the molecular level due to their thermodynamic linkage [28, 29, 57]. Simply put, a single ODC does not describe absence of the Bohr effect. Therefore, when a second ODC at lower pH is plotted to the right of a single ODC to illustrate the Bohr effect, this shift visually represents only the effect of the additional protons, not those already accounted for in the initial curve. As Malte et al. aptly note, “the Bohr effect is already built into the ODC” [28, 29]. The results presented here appear to align with this, showing that the Bohr effect is finely integrated into the ODC by the pre-loaded H^+^ to trigger the Bohr shift when reaching the inflection point of the in vitro ODC. By this triggering of the Bohr shift, Hb would be able to ensure O_2_ unloading to the tissues even though the slope of the in vitro ODC becoming shallower.

A possible interpretation can be framed through Le Chatelier’s principle. Given the six biochemical reactions underlying the Dash et al. 2016 model, O_2_ unloading depends on O_2_HbNH_2_ dissociating into HbNH_2_ and O_2_. Therefore, O_2_, HbNH_2_, or both must be sufficiently low, and/or O_2_HbNH_2_ sufficiently high, to keep Eq. (4) left leaning in accordance with tissue demand:

17 $${O}_{2}+{HbNH}_{2}\leftharpoonup {{O}_{2}HbNH}_{2}$$

During early exercise (samples 1–4, up to ≈30% $\dot{\text V}\text{O}_2$max), HbNH_2_ increases (Fig. 5A) while O_2_HbNH_2_ and the other O_2_-bound Hb species decline (Fig. 5C,D,F,H), driving Eq. (17) towards equilibrium or even reversal. To prevent this, RBCs can clear HbNH_2_ via CO_2_ binding as per Eq. (2):

18 $${CO}_{2}+{HbNH}_{2}\rightharpoonup HbNHCOOH\rightharpoonup {HbNHCOO}^{-}+{H}^{+}$$

and H^+^ binding via Eq. (5):

19 $${{HbNH}_{3}}^{+}\leftharpoonup {HbNH}_{2}+ {H}^{+}$$

In this early exercise phase, CO_2_ binding (Eq. 18) still exceeds HbNH_3_^+^ formation (Fig. 3A,C). Therefore, HbNH_2_ clearance is primarily accomplished via CO_2_ binding with HbNH_3_^+^ formation supporting. This likely reflects the continuous rise in PCO_2_ and only modest early decrease in pH (Fig. 2B,C). When O_2_Hb saturation falls below the in vitro ODC inflection point, the progressive pH drop (Fig. 2C) enhances H^+^ availability so that HbNH_3_^+^ formation supersedes CO_2_ binding, thereby replacing CO_2_ binding as the primary sink for HbNH_2_ (Fig. 3A,C). The reason for this switch appears to be that HbNH_2_ clearance by CO_2_ binding (Eq. 18) is approaching its limit as HbNHCOOH reacting to HbNHCOO^−^ slows down to eventually reverse as can be seen by the continued increase in HbNHCOOH (Fig. 5G) while HbNHCOO^−^ peaks and eventually declines with increasing $\dot{\text V}\text{O}_2$max (Fig. 5E):

20 $${CO}_{2}+{HbNH}_{2}\rightharpoonup HbNHCOOH\leftharpoonup {HbNHCOO}^{-}+{H}^{+}.$$

Together, the dominating HbNH_3_^+^ formation and partially slowing Eq. (18) can sink HbNH_2_ enough to steepen the in vivo ODC (Fig. 3A,C) between data point 4 and 7 (≈30 to 64% $\dot{\text V}\text{O}_2$max). After data point 7 (64% $\dot{\text V}\text{O}_2$max) the right-hand side of Eq. (18) finally reverses to build HbNHCOOH (Eq. 20), which slows down HbNH_2_ clearance by CO_2_ binding thereby compromising O_2_HbNH_2_ dissociation leading to the in vivo ODC inflection point. The consequential compromised tissue oxygenation then causes glycolysis to produce lactate and H⁺. This H⁺ drives HbNH_2_ protonation forward (Eq. 19, Fig. 5B), thus keeping Eq. (17) left leaning to preserve O_2_ release as much as possible, and which also activates the HCO_3_^−^ buffer to support (Fig. 5I). In short, during incremental exercise, a sequential buffering cascade, first by CO_2_ binding, then by protonation, and finally by glycolysis‐generated H⁺, continuously clears rising HbNH_2_ to keep O_2_HbNH_2_ dissociation driven towards O_2_ release, even as the ODC progressively flattens.

While this interpretation indicates compromised O_2_Hb dissociation, it seems compatible with the current lactate shuttle understanding, whereby lactate is not a consequence of an O_2_ debt but a marker of physiological and metabolic strain [7, 8, 38]. Instead of accumulating because of an O_2_ deficit, lactate production signals the metabolic strain invoked to sustain tissue oxygenation when O_2_ unloading is challenged by the convex part of the ODC. In addition, these findings seem to support the work by Kobayashi et al., who published a few, if not the only existing, explicit articles on the inflection point of the ODC [22–24, 26]. The authors back then used the Adair equations to calculate the ODC and emphasized that “the steep portion of the OEC [oxygen equilibrium curve = ODC] around *S* [${S}_{{HbO}_{2}}$] = 0.38 is exploited for large oxygen demands under conditions of exercise. The most efficient O_2_ unloading region around S = 0.38” [22].

Basic science continues to uncover ever new details about the Bohr effect all the way down to the atomic level of Hb [59, 61]. Such insights are essential, as manipulating this mechanism is key to treating various medical conditions. As reviewed by Pagare et al. [36], drugs stabilizing the tense state of Hb to enhance O_2_ delivery show therapeutic potential for ischemia-related cardiovascular conditions like angina, myocardial ischemia, stroke, and trauma [17, 18, 20, 25, 42, 50], while relaxed state stabilizers, increasing Hb’s O_2_ affinity, offer promise for sickle cell disease [1–4, 15, 31–35, 40, 51, 54, 58, 60]. Additionally, maintaining Hb’s original functionality is crucial for developing Hb-based O_2_ carriers that ensure proper physiological performance [11, 43]. The built-in Bohr effect, the intersection of CO_2_ and HbNH_3_^+^, and the following alignment of the in vivo inflection points with the critical capillary PO_2_ might be useful in guiding such developments.

Lastly, the inherent Bohr effect in a single ODC and its instrumental importance in setting the shape and position of the ODC, as phrased by Malte et al. [28, 29], may explain the fundamental issues of nH to quantify the cooperative binding to Hb. When an ODC (Hill equation) is linearized to create a Hill plot, the slope of which is determined to derive nH, it follows that nH reflects both the cooperativity and the Bohr effect.

The results of our analysis must be understood within the limits of Dash et al.’s model of the ODC, CDC, and HDC. In particular, the model does not account for the blood-tissue transport of O_2_, CO_2_, HCO_3_^−^, and H^+^ in microcirculation and buffering CO_2_ in blood and tissue, as this is addressed in another model by the authors [12, 13]. Another limitation is that the data of Stringer et al. are the mean values of five participants. Therefore, the inputs for PO_2_, PCO_2_, pH, and the measured saturation are subject to a certain degree of uncertainty, for which we accounted by fitting those data and running the fitted values through the Dash et al. model as well. Also, while Hct and Hb levels in the Dash et al. model do not affect the calculated ODC, CDC, and HDC curves, it does, of course, affect the absolute amounts of O_2_, CO_2_, and H^+^ bound. While this needs to be considered, it induces only an offset error, which has no effect on the location of the inflection points or the intersection of CO_2_ and H^+^. We considered the ± 4 percentage point limit of agreement for O_2_Hb saturation acceptable, as most data points, especially those in the middle of the data set, fell within ± 2 percentage points, and our sensitivity analysis confirmed that the results were robust to changes in temperature.

In conclusion, this mathematical model simulation-based physiological data processing and analysis showed that the Bohr shift is a finely tuned mechanism embedded within the ODC itself. It is triggered at the in vitro ODC inflection point, where HbNH_3_^+^ formation supersedes CO_2_ binding to HbNH_2_, thereby steepening the in vivo ODC. This steepening sustains O_2_ unloading until it reaches its limit, marked by the in vivo ODC inflection point, which aligns with the GET. These findings provide a novel direct mechanistic link between Hb function and the GET, highlighting its physiological relevance. Prospective experimental studies should be carried out to investigate these mechanisms and their implications for clinical applications.

## Data Availability

The complete dataset, including pooled mean femoral vein samples (n = 12) with %$\dot{\text V}\text{O}_2$max at sampling, PO_2_, PCO_2_, pH, and O_2_Hb saturation, along with the full analysis code (R programming language) used to produce the findings and Figs. 2–5 is freely available in the following repository: 10.5281/zenodo.15614693
